# Exploring the complexity of commonly held attitudes and beliefs of low back pain—a network analysis

**DOI:** 10.3389/fmed.2024.1327791

**Published:** 2024-01-24

**Authors:** Bernard X. W. Liew, Ben Darlow

**Affiliations:** ^1^School of Sport, Rehabilitation and Exercise Sciences, University of Essex, Colchester, United Kingdom; ^2^Department of Primary Healthcare and General Practice, University of Otago, Wellington, New Zealand

**Keywords:** low back pain, psychological factors, beliefs, network analysis, attitudes

## Abstract

**Objectives:**

The current study used a network analysis approach to explore the complexity of attitudes and beliefs held in people with and without low back pain (LBP). The study aimed to (1) quantify the adjusted associations between individual items of the Back Pain Attitudes Questionnaire (Back-PAQ), and (2) identify the items with the strongest connectivity within the network.

**Methods:**

This is a secondary data analysis of a previously published survey using the Back-PAQ (*n* = 602). A nonparametric Spearman’s rank correlation matrix was used as input to the network analysis. We estimated an unregularised graphical Gaussian model (GGM). Edges were added or removed in a stepwise manner until the extended Bayesian information criterion (EBIC) did not improve. We assessed three measures of centrality measures of betweenness, closeness, and strength.

**Results:**

The two pairwise associations with the greatest magnitude of correlation were between Q30–Q31 [0.54 (95% CI 0.44 to 0.60)] and Q15–Q16 [0.52 (95% CI 0.43 to 0.61)]. These two relationships related to the association between items exploring the influence of attentional focus and expectations (Q30–Q31), and feelings and stress (Q15–Q16). The three items with the greatest average centrality values, were Q22, Q25, and Q10. These items reflect beliefs about damaging the back, exercise, and activity avoidance, respectively.

**Conclusion:**

Beliefs about back damage, exercise, and activity avoidance are factors most connected to all other beliefs within the network. These three factors may represent candidate targets that clinicians can focus their counseling efforts on to manage unhelpful attitudes and beliefs in people experiencing LBP.

## 1 Introduction

Low back pain (LBP) is a highly prevalent and costly musculoskeletal pain disorder, which occurs in all countries and affects individuals across the lifespan (1). In 2019, LBP accounted for over half a billion prevalent cases and over 60 million years lived with disability (YLDs) (1). In 2016, LBP was ranked first in healthcare expenditure of US$134.5 billion in the United States (2). Two-thirds of those with an acute painful episode of LBP recover within the first three months (3), although relapses and remissions are common (4). Approximately 20% of LBP sufferers go on to experience severe, high-impact chronic pain (5), which contributes most to healthcare costs.

The attitudes and beliefs of both patients and clinicians are thought to be important contributors to the development of LBP, its recovery, and how the condition is managed. Harboring negative attitudes and beliefs regarding LBP may elevate catastrophic thoughts and avoidant behaviors (6), which cascade into greater disuse and depression (7), leading to delayed recovery and functional return (8, 9). The beliefs of healthcare practitioners have been reported to explain as much as 20% of the variance in their recommendations to patients suffering from LBP (10). The importance of the relationship between beliefs and clinical management has also been reported across many healthcare professions and in different countries (11). To measure the attitudes and beliefs regarding LBP, the 34-item Back Pain Attitudes Questionnaire (Back-PAQ) was developed (12). Further work on the Back-PAQ resulted in the development of an abbreviated 20-item (13) and also a 10-item version (12).

Regardless of the versions of the Back-PAQ, a summative total score is determined by aggregating the values across all items (12). For example, the original Back-PAQ has a total score ranging from 34 to 170, with higher scores indicating more negative beliefs regarding LBP (12). This aggregate score has been used in clinical trials to determine the effects of different interventions on changes in the patient’s attitudes and beliefs (14). Analyzing only the aggregate score of the Back-PAQ does not fully maximize the use of the information. This is because two individuals can have similar aggregate Back-PAQ scores, but have very different scores on individual items. Determining the most important Back-PAQ items could improve its utility for clinical decision-making.

There are potentially many approaches in seeking to understand the most important facets underlying an individual’s beliefs about LBP. In a previous study of the Back-PAQ in the general population, items relating to posture (Q8), muscle strength (Q7), and lifting technique (Q5) were the most negatively scored (6); it may be that items with the worse score are considered the most important items. Patients and clinicians are commonly thought to hold negative beliefs about the safety of certain lifting postures (15, 16), and the appropriateness of physical activity during an episode of LBP (17). The importance of the beliefs about posture and activity resumption is evidenced by the development of therapeutic interventions seeking to target these specific beliefs (18, 19). Alternatively, facets of an individual’s belief system with strong prognostic value may be deemed as important, including expectations about recovery (20), and self-efficacy (21).

Another approach to determining the importance of items is via network analysis (22). Network analysis focuses on quantifying the multivariate relationships between individual items (23, 24). The importance of any item in network analysis, also termed centrality, is typically defined by the magnitude of association, and the closeness of associations to all other items (22). An item with a very high score may not be central, if it is connected to very few items. In a hypothetical scenario of negative beliefs about posture, a low centrality would mean that this specific belief does not affect the beliefs on other items. From a treatment perspective, targeting a low central item would not be the most efficient approach.

The current study explored the multivariate relationship of the items within the Back-PAQ. The main aims of the study were to (1) describe the network and identify the item pairs with the strongest adjusted associations, and (2) identify a reduced set of items with the strongest connectivity within the network. Given that network analysis is a data-driven approach, and that this is the first study to apply such techniques on the Back-PAQ, there were no priori hypotheses made about what item pairs would be the most correlated, or which items would have the greatest centrality measures.

## 2 Methods

### 2.1 Study design

Secondary data analysis using the methodology of network analysis.

### 2.2 Participants

This study used data from a previously published survey of the New Zealand population that used the Back-PAQ (6). One thousand people who were 18 years and older were randomly selected from the New Zealand Electoral Roll and invited by mail to complete the survey. The survey was completed by 602 participants (female = 331, male = 271). Participant characteristics are described in detail in the original publication (6), but briefly, 76 participants self-reported never having experienced a back pain history, 361 reported a past experience of a back pain history, 164 reported a current experience a back pain history, and one participant did not self-report.

### 2.3 Questionnaire

The Back-PAQ is a 34-item questionnaire (Table 1), scored on a a five-level ordinal scale [responses coded from “False” = 1 to “True” = 5 (Table 1)]. Eleven items (1, 2, 3, 15, 16, 17, 27, 28, 29, 30, 31) are reversed compared with the normal direction of the survey. Hence, for these 11 items, the answers were re-coded with the normal direction of the survey. The total score range from 34 to 170, with a higher score reflecting more unhelpful beliefs. The Back-PAQ has acceptable internal consistency (*α* = 0.70) (12), excellent test-retest reliability (ICC = 0.84) (25), and moderate convergent validity relative to the Tampa Scale of Kinesiophobia (*r* = −0.58) (25) when used by a cohort of healthcare practitioners.

**Table 1:** Individual items and their questions of the Back-PAQ.

Items	Question
1	Your back is one of the strongest parts of your body
2	Your back is well designed for the way you use it in daily life
3	Bending your back is good for it
4	Sitting is bad for your back
5	Lifting without bending the knees is not safe for your back
6	It is easy to injure your back
7	It is important to have strong muscles to support your back
8	Good posture is important to protect your back
9	If you overuse your back, it will wear out
10	If an activity or movement causes back pain, you should avoid it in the future
11	You could injure your back if you are not careful
12	You can injure your back and only become aware of the injury sometime later
13	Back pain means that you have injured your back
14	A twinge in your back can be the first sign of a serious injury
15	Thoughts and feelings can influence the intensity of back pain
16	Stress in your life (financial, work, relationship) can make back pain worse
17	When you have back pain, you can do things which increase your pain without harming the back
18	Having back pain makes it difficult to enjoy life
19	It is worse to have pain in your back than your arms or legs
20	It is hard to understand what back pain is like if you have never had it yourself
21	If your back hurts, you should take it easy until the pain goes away
22	If you ignore back pain, you may cause damage to your back
23	It is important to see a health professional when you have back pain
24	To effectively treat back pain you need to know exactly what is wrong
25	If you have back pain you should avoid exercise
26	When you have back pain the risks of vigorous exercise outweigh the benefits
27	If you have back pain you should try to stay active
28	Most back pain settles quickly, at least enough to get on with normal activities
29	Worrying about your back can delay recovery from back pain
30	Focussing on things other than your back helps you to recover from back pain
31	Expecting your back pain to get better helps you to recover from back pain
32	Once you have had back pain there is always a weakness
33	There is a high chance that an episode of back pain will not resolve
34	Once you have a back problem, there is not a lot you can do about it

### 2.4 Approach to network analysis

#### 2.4.1 Software and packages

The dataset was analyzed with R statistical software (version 4.2.2). Several packages were used to perform the analyzes, including *qgraph* (26) for network estimation and plotting, and *bootnet* (27) for stability analysis. Since the Back-PAQ items are ordinal, a nonparametric Spearman’s rank correlation matrix was used as input to the network analysis. We estimated an unregularised Graphical Gaussian model (GGM), using the *ggmModselect* algorithm with the following parameters (28): tuning = 0.25, stepwise = TRUE, consider PerStep = “subet,” and missing = “pairwise.” From a graphical lasso network model, edges were iteratively added and removed until the extended Bayesian information criterion (EBIC) did not improve (28). This is similar to performing stepwise selection in regression models using Akaike information criterion.

Presently, we assessed three measures of centrality: betweenness (how often one node lies on the shortest path between other nodes), closeness (shortest edges to other nodes), and Strength (magnitude of all the node’s immediate edges) (29). Clinically, a node high in Strength can directly influence many adjacent nodes, without the influence of other nodes (29). A node high in Closeness can be interpreted as the speed of influence a change in one node has on all other nodes in the network (29). Lastly, if a node high on Betweenness were to be removed, the relationship between all other nodes become more indirect (29).

We assessed the variability of the edge weights using bootstrapping (*B* = 1,000) (27), to estimate the 95% confidence interval of the estimated edge weights (i.e., the partial correlations). To gain an estimate of the variability of the found centrality indices (CS-coefficient)—meaning if the order of centrality indices remains the same after re-estimating the network with fewer participants, we applied the participant-dropping subset bootstrap (*B* = 1,000) (27). This procedure drops a percentage of participants, re-estimates the network, and re-calculates the three centrality indices. The percentage of participants dropped ranged from 5% to 75%, across 10 sampling levels. The CS-coefficient reflects the maximum proportion of participants that can be dropped, such that with 95% probability the correlation (of the centrality value of the bootstrapped sample vs. that of the original) would reach a certain value (0.7 in the current study, CS_cor = 0.7_). It is suggested that the CS_cor = 0.7_ should be >0.25 and is better if it is >0.5 (27).

## 3 Results

**Figure 1:**
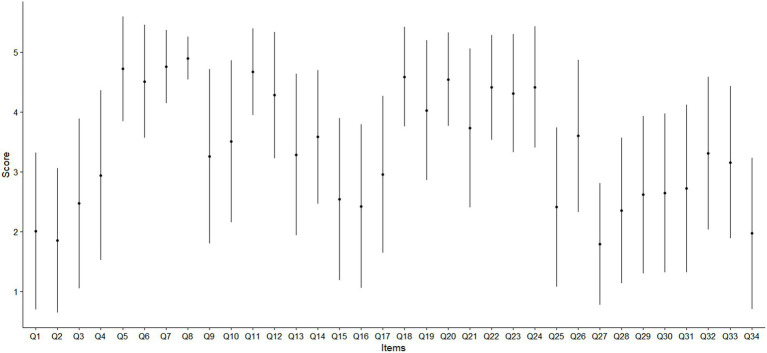
Mean and error bar as one standard deviation of the cohort’s individual item score of the Back-PAQ.

**Figure 2:**
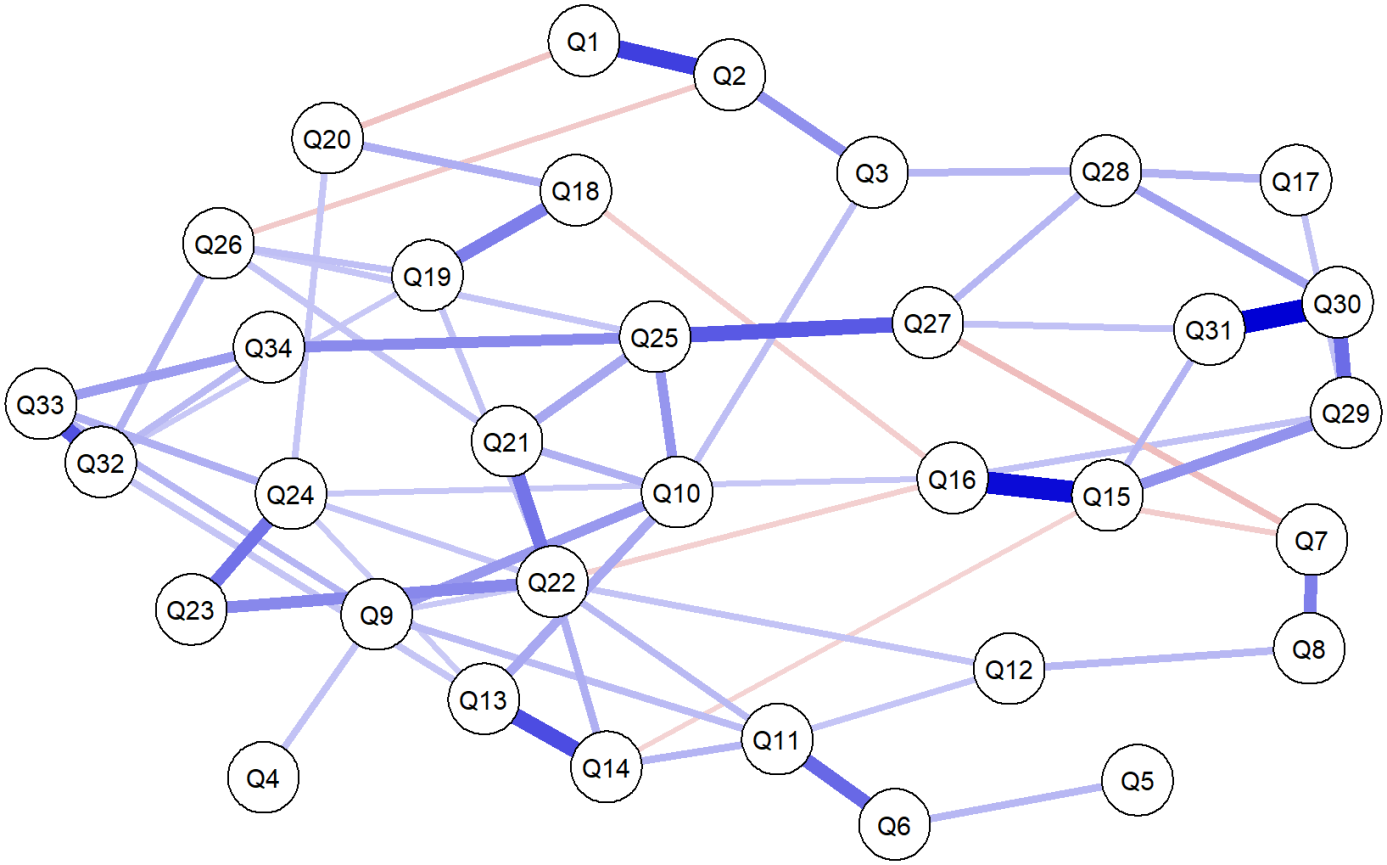
Network analysis of the association between the 34 items of the Back-PAQ. Each edge in the network represents either positive regularized adjusted associations (blue edges) or negative regularized adjusted associations (red edges). The thickness and color saturation of an edge denotes its weight (the strength of the association between two nodes).

**Figure 3:**
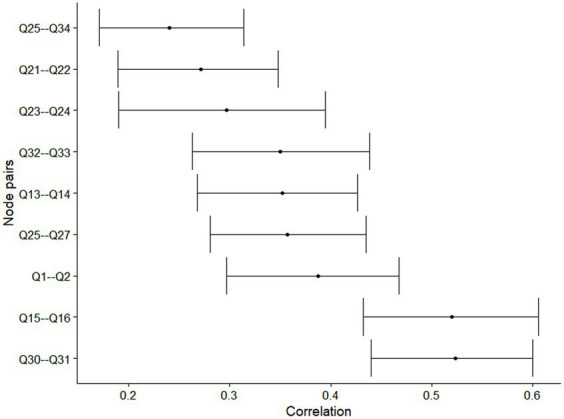
Bootstrapped 95% confidence interval of the estimated edge weights of the network. Only edges where 100% of the bootstrapped estimated correlation was non-zero retained for plotting.

The mean (standard deviation) score of each item of the Back-PAQ can be found in Figure 1. The five pairwise associations with the greatest magnitude of correlation were between Q30–Q31 [0.54 (95% CI 0.44 to 0.60)], Q15–Q16 [0.52 (95% CI 0.43 to 0.61)], Q1–Q2 [0.41 (95% CI 0.30 to 0.47)], Q13–Q14 [0.38 (95% CI 0.27 to 0.43)], and between Q32–Q33 [0.37 (95% CI 0.26 to 0.44)] (Figures 2, 3). These five relationships related to the association between items exploring the perceived influence of attentional focus and expectations (Q30–Q31), items exploring the perceived influence of feelings and stress (Q15–Q16), items exploring the strength and design of the back (Q1–Q2), items exploring interpretations of pain and injury (Q13–Q14), and items exploring persistent weakness and pain (Q32–Q33) (Table 1).

**Figure 4:**
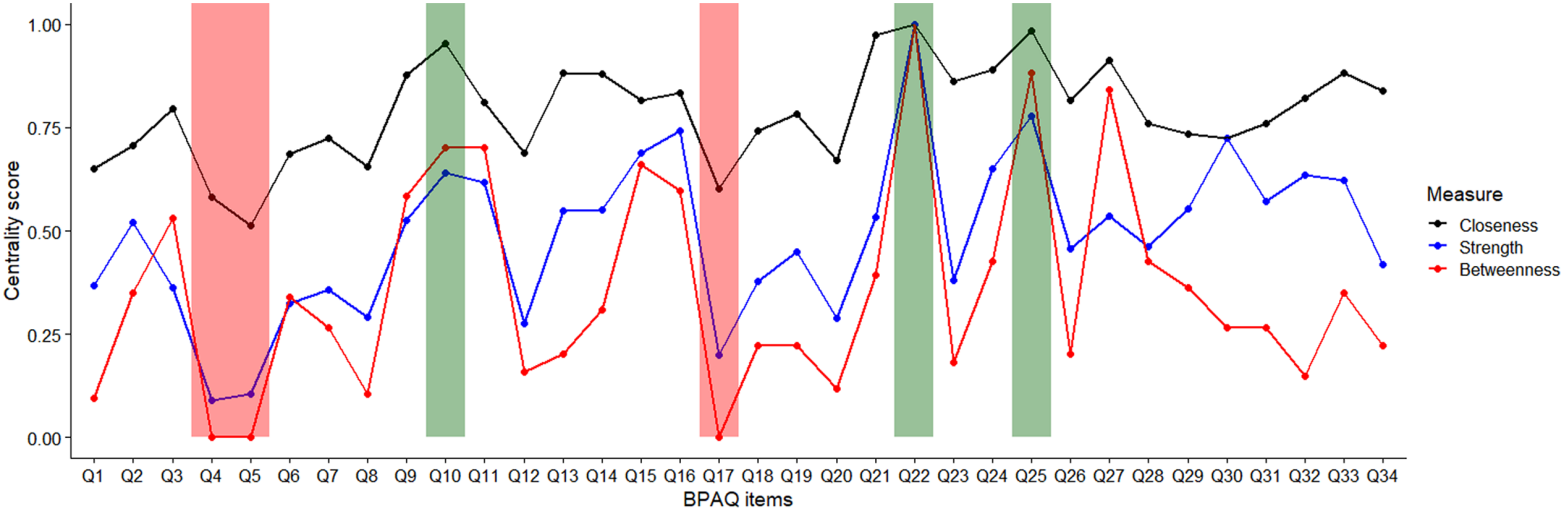
Standardized (0–1) centrality scores of the 34 items of Back-PAQ. Items shaded in green indicate the top three most central items and those in red indicate the least central items.

The three nodes with the greatest average centrality values across betweenness, closeness, and strength, were Q22 (betweenness = 1.00, closeness = 1.00, strength = 1.00), Q25 (betweenness = 0.88, closeness = 0.98, strength = 0.78), and Q10 (betweenness = 0.70, closeness = 0.95, strength = 0.64) (Figure 4). The three nodes with the lowest average centrality values across strength, betweenness, and closeness, were Q4 (betweenness = 0.00, closeness = 0.58, strength = 0.09), Q5 (betweenness = 0.00, closeness = 0.51, strength = 0.11), and Q17 (betweenness = 0.00, closeness = 0.60, strength = 0.20) (Figure 4). The stability of the centrality measures, CS_cor = 0.7_, of betweenness, closeness, and strength were 0.05, 0.00, and 0.60, respectively.

## 4 Discussion

Attitudes and beliefs are thought to be important contributors to the development, recovery, and management of LBP. The present study aimed to understand the complex relationship between the individual items of the Back-PAQ to better understand how different beliefs interact with each other. The top two most correlated edges were between focus and expectations (Q30–Q31), and feelings and stress (Q15–Q16). In addition, the three items with the greatest average centrality values across betweenness, closeness, and strength, were Q22, Q25 and Q10. These items reflect beliefs about damaging the back, exercise and activity avoidance, respectively.

A recent systematic review have reported that recovery expectations is a prognostic factor of return to work and recovery outcomes (20), and that expectation of symptom change modulates changes in pain and impairment (30, 31). From our network analysis, a more positive belief about recovery expectations was associated with more positive beliefs about the benefits of focusing on things other than the back (Q30), staying active (Q27), and acknowledging the role of thoughts and feelings in LBP (Q15). These associations may represent candidate mechanisms by which recovery expectations influence LBP outcomes. From the literature, it is thought that recovery expectations might affect LBP outcomes by modifying coping, healthcare-seeking, and withdrawal behaviors (20). It may be that with a more positive belief about focusing on other things and staying active, patients have greater self-efficacy in pursuing activities, despite the presence of pain, which ultimately benefits the recovery of LBP.

Even though beliefs about good posture (Q8) were not most correlated with having strong muscles (Q7), our findings still support their direct association. The present finding supports prior research which reported that patients frequently viewed correct lifting techniques, posture, and having strong muscles as collective strategies for protecting the back (32). Interestingly, beliefs about bending (Q3), sitting (Q4), and lifting (Q5), were not directly associated with each other (Figure 1). Some of these beliefs have been thought to have their roots in communication with clinicians (32) and mass media campaigns (33). If beliefs about bending, sitting, and lifting had a common cause, it would be likely that they are directly associated with each other. Findings from the present study suggest that each of these three beliefs may not be as closely associated as previously thought (34), and may have different antecedent causes. Clinically, this suggests that if educational efforts were to be directed toward altering the beliefs of these activities, they will have to be done so individually, rather than with the expectation that changing the beliefs on one task will influence another.

Items on the Back-PAQ with the worse score may not always be the most connected items within the network. For example, items relating to posture (Q8), muscle strength (Q7), and lifting technique (Q5) were the most negatively scored (6), but represented some of the least central items (Figure 4). In other words, these aforementioned items are relatively isolated from all other items. The most central items relate to beliefs about causing back damage (Q22), the benefits of avoiding exercise (Q25), and activity avoidance (Q10). These three beliefs have close relations with prior reported perceived myths about LBP, particularly on the role of tissue damage in LBP, and the importance of stopping exercise and activity when LBP occurs (34). Not surprisingly, these unhelpful beliefs about exercise are also held by clinicians [e.g., Q9 in (35)], reinforcing the importance of the enduring influence of clinical opinions on the beliefs of LBP on lay people (32). Prior qualitative research has reported that negative beliefs about low back tissue damage results in high pain-related fear (36), while quantitative longitudinal research have reported that fear is a prognostic indicator of persistent LBP symptoms (9). Our findings also support prior research which identified that LBP individuals with high pain-related fear have two predominant beliefs—the potentially damaging effects of physical activity and that performing an activity with pain will increase suffering (37).

The network visualization is clinically very intuitive, enabling rapid and unique clinical insights which may be used to efficiently guide patient counseling. For example, our findings showed that the belief about the ease of injury (Q6) is directly associated with the belief about the safety of lifting (Q5), and not sitting (Q4). This means that for clinicians desiring to alter a patient’s beliefs about sitting safety, educational efforts to modify the patient’s beliefs about the vulnerability of the spine to injury may not be the most efficient treatment approach. Second, in a busy clinical environment, findings from the present study suggest that educational efforts should focus on targeting beliefs related to back damage (Q22), exercise, and activity avoidance (Q25 and Q10). A recent editorial published the 10 common myths about LBP, calling on clinicians to incorporate these discussions with their patients (34). The present finding supplements prior clinical recommendation reports (34), providing evidence for the most efficient approach to navigating these beliefs with patients.

This study has several limitations. First, no attempt was made to distinguish the network dynamics of the Back-PAQ among people with and without LBP. Future investigations on understanding the differences in belief systems among different LBP subgroups may be useful for personalizing education efforts in managing and preventing LBP. Second, the longitudinal relationship between individual items of the Back-PAQ and clinical outcomes was not investigated. Including both the items of the Back-PAQ and measures related to clinical outcomes (e.g., pain intensity and impairment at follow-up) in a prospective study, may help to identify specific beliefs driving clinical outcomes. Third, the original study recruited participants with and without LBP randomly selected from an Electoral Roll. Information concerning the duration of current LBP and whether LBP had a specific cause (e.g., spondyloarthropathy), was not collected. A previous study reported that individuals with axial spondyloarthropathy reported lesser LBP intensity and better health related quality of life, than those with chronic non-specific LBP (38). Whether similar attitudes and beliefs are held in people with specific and non-specific LBP remains to be investigated.

## 5 Conclusion

Network analysis of the Back-PAQ revealed unique insights into the beliefs about LBP. Beliefs about back damage, exercise, and activity avoidance are factors most connected to all other beliefs within the network. This suggests that these three factors represent candidate targets that clinicians can focus their patient counseling efforts on.

## Data availability statement

The raw data supporting the conclusions of this article will be made available by the authors, without undue reservation.

## Ethics statement

The studies involving humans were approved by This study was approved by the University of Otago Ethics Committee (D12/255). The studies were conducted in accordance with the local legislation and institutional requirements. The participants provided their written informed consent to participate in this study.

## Author contributions

BL: Conceptualization, Formal analysis, Methodology, Software, Validation, Visualization, Writing – original draft, Writing – review & editing. BD: Conceptualization, Data curation, Funding acquisition, Investigation, Methodology, Project administration, Resources, Supervision, Validation, Writing – original draft, Writing – review & editing.
